# Information Value-Based Fault Diagnosis of Train Door System under Multiple Operating Conditions

**DOI:** 10.3390/s20143952

**Published:** 2020-07-16

**Authors:** Seokgoo Kim, Nam Ho Kim, Joo-Ho Choi

**Affiliations:** 1 Department of Aerospace & Mechanical Engineering, Korea Aerospace University, Goyang-City 10540, Korea; sgkim@kau.kr; 2 Mechanical & Aerospace Engineering, University of Florida, Gainesville, FL 32611, USA; nkim@ufl.edu; 3 School of Aerospace & Mechanical Engineering, Korea Aerospace University, Goyang-City 10540, Korea

**Keywords:** multiple classifier, Bayesian network, multiple operating conditions, train door system, information value

## Abstract

While there are many data-driven diagnosis algorithms for fault isolation of complex systems, a new challenge arises in the case of multiple operating regimes. In this case, the diagnosis is usually carried out for each regime for better accuracy. However, the problem is that different results can be derived from each regime and they can conflict with each other, which may invalidate the performance of fault diagnosis. To address this challenge, a methodology for selecting the most reliable one among the different diagnostic results is proposed, which combines the Bayesian network (BN) and the information value (IV). The BN is trained for each regime and a conditional probability table is obtained for probabilistic fault diagnosis. The IV is then employed to evaluate the value of several diagnostic results. The proposed approach is applied to the fault diagnosis of a train door system and its effectiveness is proven.

## 1. Introduction

Health diagnostics of mechanical systems and remaining useful life (RUL) prediction brings numerous benefits such as safety system operation, zero downtime, cost-effective maintenance scheduling. To realize these aspects, many studies have been conducted under the name of prognostics and health management (PHM). There are several review papers that address the recent research trend of PHM [1,2,3]. Basically, PHM can be grouped into two main aspects: fault diagnosis and prognosis. Diagnosis is the prior stage of prognosis because accurate fault isolation and fault severity estimation are directly related to the accuracy of prognostics. Most of the fault diagnostics approaches can be categorized into the model-based and data-driven method [4]. In the case of model-based methods, users are required to establish mathematical models of the system based on the physics of failure, in which the physical parameters are estimated from the sensors data [5]. Data-driven approaches use large amounts of training datasets to train machine learning algorithms to diagnose the health state of the system [6]. Recently, deep learning algorithms are gaining popularity as an alternative option in the data-driven diagnostics approach due to less involvement of features processing [7,8,9,10]. Each approach has its own pros and cons. Model-based methods are superior in terms of accuracy. However, it is rarely possible to establish such a model. Data-driven approaches are more common in the field, but require a large amount of data that is not easily available in the industry [11,12]. Users should choose a proper one based on their environments for effective PHM implementation.

In the railway system, the passenger access system (PAS) is known to operate under highly stressed conditions over time and is regarded as one of the most critical parts in the view of safety. PAS is responsible for 30–40% of the failures during operation [13]. In order to prevent such failures, model-based and data-driven approaches have been applied to the fault diagnostics of the PAS. In the model-based approach, Bond Graph modeling of a train door is employed to carry out a global FDI (Fault Diagnostics and Isolation) for the fault indicators and residual threshold in the presence of door failures [14]. Lin et al. established a mathematical model of an urban train door system to estimate physical parameters in the case of normal and faulty conditions [15]. Then, the principal component analysis (PCA) is applied to perform the fault diagnosis. Dassanayake et al. performed fault detection and diagnosis of an electric train door by parameter estimation of the system model [16]. In the knowledge-based and data-driven approach, a Petri net behavioral model, which includes normal and faulty condition operating, is established, which is used for fault diagnosis of PAS [17]. Similar to the train door system, Yan and Lee used information gathered from controllers or sensors in the elevator door system and performed on-line performance degradation assessment and root cause analysis using multiple logistic regression (LR) [18]. Apart from these approaches, there have been continued studies employing the Bayesian network (BN) for the fault diagnosis, which is a probabilistic causal network that represents a set of random variables and their conditional dependencies. For decades, it has been widely applied in numerous domains from reliability engineering, risk analysis, and medical diagnosis [19]. In the fault diagnosis, there have been several approaches using BN. For example, Yang and Lee applied BN to predict the wafer quality of a semiconductor manufacturing system and inferred which sensors are directly related to the wafer quality [20]. Xu et al. performed fault inference for rotating flexible rotors with an attempt to enhance the reasoning capacity under conditions of uncertainty with BN [21]. Cai et al. applied PCA and used the principal components as the input nodes of BN for the fault diagnostics of a three-phase inverter [22]. Zheng et al. combined fault tree (FT) and BN to diagnose the bridge crane spreader. This method proved that the proposed method could reduce the amount of required data for model training by using prior knowledge for the system [23]. More applications of the BN in the fault diagnosis can be found in [19].

In this study, the BN is applied to the train door system for the purpose of fault diagnosis using the motor current signals acquired during the door operation. Although there exists literature with the similar applications, a new challenge arises in this problem, which is the issue of multiple operation stages, namely, the train door moves under three different conditions: acceleration, constant motion, and deceleration. As will be discussed in the main text, the diagnosis performance is significantly affected by whether the velocity stages are considered separately or not. In order to achieve better results, it is more advantageous to divide the current signal into different stages, and training is performed respectively.

The problem is, however, that the algorithm can yield different diagnosis results in each stage, which can confuse the identification of the fault modes. Several methods have been proposed to deal with the issue of different or competing results from multiple classifications. Zhang et al. combined multiple neural networks to obtain a more reliable diagnosis than a single one by using the modified majority voting method [24]. The result was compared with original majority voting, averaging, and weighted averaging. Niu et al. proposed a decision fusion for fault diagnosis that integrates data from different types of sensors and decisions of multiple classifiers [25]. The multiple classifiers are fused by using a multi-agent combination algorithm. W. Yan and Xue introduced a dynamic fusion approach and applied it to an aircraft engine fault diagnosis [26]. Their performance was compared with other fusion approaches such as simple averaging and local accuracy-based selection. Existing pieces of literature, however, have focused only on the fusion of different algorithms trained by the data under the same operating condition. On the other hand, this study aims at the fusion of different diagnosis results by multiple operation data using a single BN algorithm.

To solve this problem, this paper proposes a new method that introduces information value (IV) based on the training data to suggest the most reliable classifier. The paper is organized as follows. Section 2 introduces the theoretical background of the Bayesian network. In Section 3, the basic concept of IV is explained. Application to the train door system is introduced in Section 4 and finally, the paper is concluded in Section 5.

## 2. Bayesian Network

Bayesian network (BN) is a probabilistic graphical model which represents conditional dependencies or causal connections between a set of random variables via a Directed Acyclic Graph (DAG). BN is capable of reasoning under uncertainty, where the nodes represent variables (discrete or continuous) and links represent direct connections between them. In addition, BN models the quantitative strength of the connections between variables, allowing probabilistic beliefs about them to be updated automatically as new information becomes available [27]. The BN-based fault diagnosis consists of three steps: (1) Determine the network structure, (2) establish the conditional probability table (CPT), and (3) carry out probabilistic fault diagnosis based on given evidence. In the BN, the DAG is called the structure and the values in the CPT are called the parameters.

### 2.1. Basis of Bayesian Network

**Figure 1:**
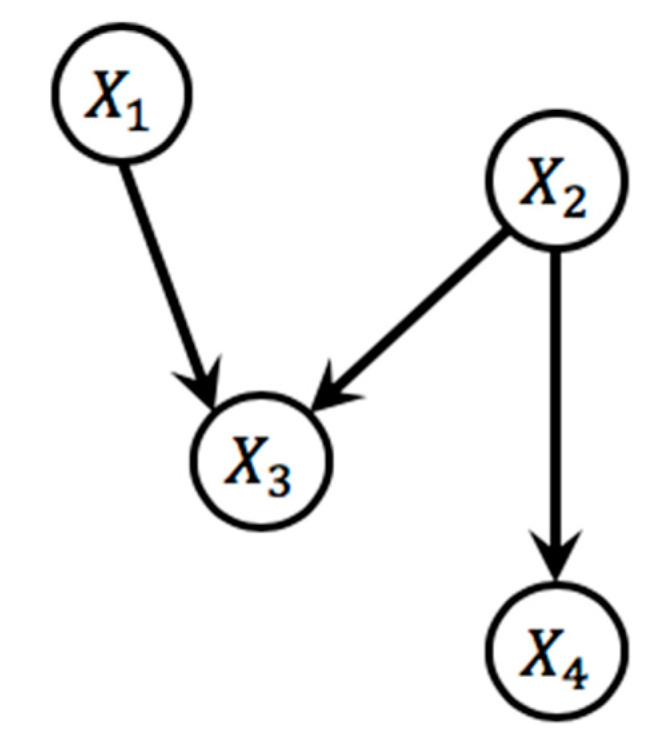
An example of a Bayesian network.

Let us assume a network model which consists of four nodes named $X_{1},X_{2},X_{3},$ and $X_{4}$. The joint probability of the illustrated model can be written as

(1) $$P\left(X_{1},X_{2},X_{3},X_{4}\right)=P\left(X_{1}\right)P(X_{2}|X_{1})P(X_{3}|X_{1},X_{2})P(X_{4}|X_{1},X_{2},X_{3})$$

where $2^{4}-1=15$ conditional probability parameters are required to construct the full joint probability when each node has binary status. On the other hand, the BN assumes conditional independence which leads to the reduction of the required number of parameters to calculate joint probability. In the network model shown in Figure 1, $X_{2}$ is the parent node of $X_{3}$ and $X_{4}$, which are conditionally independent each other, and $X_{1}$ is non-immediate parent nodes of $X_{4}$, i.e., $P\left(X_{4}|X_{1},X_{2},X_{3}\right)=P(X_{4}|X_{2})$. Applying these relations, the joint probability can be obtained as follows

(2) $$P\left(X_{1},X_{2},X_{3},X_{4}\right)=P\left(X_{1}\right)P\left(X_{2}\right)P(X_{3}|X_{1},X_{2})P(X_{4}|X_{2})$$

where the number of parameters is now reduced to 8. Based on this, any type of probability can be calculated with joint probability.

### 2.2. Structure Learning and Parameter Learning for Bayesian Network

The first step of BN-based fault diagnosis is to establish a network structure which reflects the interconnection between random variables. In simple words, the structure implies a set of conditional independence relations among the variables involved [28]. When a domain expert or system user already understands paths of possible influence between variables or the fault tree, the structure of BN can be established based on the domain expert. In some cases, however, it is not a simple matter to find the structure of a BN. In this case, the structure can be determined automatically by applying BN learning algorithms. Among others, the score-based approach is one of the most popular methods, including the Akaike information criterion (AIC), the Bayesian information criterion (BIC), the minimum description length (MDL), and K2 [20]. This paper employs the K2-algorithm which was developed by Cooper [29] and is known as the simplest approach. The benefit of the K2 algorithm is that prior knowledge for the network structure can be embedded by defining node order in advance to reduce the unnecessary computation. Given database *D* and a candidate network structure $B_{S}$, the K2 algorithm searches the BN structure, maximizing the probability $P(B_{s}|D)$. This algorithm requires node ordering and an upper limit of the number of parent nodes as the input to reduce the computational complexity. Then, the algorithm searches the most likely set of parent nodes which precedes the current node based on the node ordering by calculating the probability of each case. In other words, it searches the set of parent nodes maximizing the following probability function:

(3) $$g\left(i,\pi_{i}\right)=\overset{q_{i}}{\underset{j=1}{\prod }}\frac{\left(r_{i}-1\right)!}{\left(N_{ij}+r_{i}-1\right)!}\overset{r_{i}}{\underset{k=1}{\prod }}N_{ijk}!$$

where i is the index of the node variable $x_{i},\pi_{i}$ is the set of its parent nodes, $q_{i}$ is the unique instantiations of the parents of *x_i_* in the database, $r_{i}$ is the number of all possible values of $x_{i}$, and $N_{ijk}$ is the number of cases in the database in which the variable $x_{i}$ has $k^{th}$ value, and the parents of *x_i_* are instantiated with the $j^{th}$ instances among all possible instantiations of the $\pi_{i}$. Note that $N_{ij}$ can be obtained by $\overset{r_{i}}{\underset{k=1}{\sum }}N_{ijk}$. Algorithm 1 illustrates the pseudo-code for the K2 algorithm and details can be found in references [29,30,31]. As a result, optimum BN structure is determined based on the K2 algorithm.
**Algorithm 1:** The K2 algorithm1: **procedure** K2;2: {Input: A set of *n* nodes, an ordering on the nodes, an upper bound *u* on the3:   number of parents a node may have, and a database *D* containing m cases.}4: {Output: For each node, a printout of the parents of the node.}5: **for**
*i* := 1 to *n* do6:  $\pi_{i}:=\emptyset ;$
7:  *P*_old_ := *g*$(i,\;\pi_{i}$);8:  OKToProceed :=
**true**9:  **while** OKToProceed and |$\pi_{i}$| < *u*
**do**10:   let *z* be the node in Pred(*x_i_*) - $\pi_{i}$ that maximizes *g*$(i,\;\pi_{i}$
$\cup^{\text{​}}$ {*z*});11:   *P_new_*
:=
*g*$(i,\;\pi_{i}$
$\cup^{\text{​}}$ {*z*});12:   **if**
*P_new_* > *P_old_*
**then**13:    *P_old_*
:= P_new_;14:    $\pi_{i}:=\;\pi_{i}\cup^{\text{​}}\left\{z\right\}$
15:   **else** OKToProceed :=
**false**;16:  **end** {while};17:  **write** (‘Node:’, $x_{i}$, ‘Parents of this node:’, $\pi_{i}$)18: **end** {for};19: **end** {K2};

Once the network structure is determined by the K2 algorithm, next is to establish the CPT. CPTs are usually obtained by two ways: domain expert’s knowledge or learning from normal and fault data [22]. In this paper, CPTs are calculated from training data by implementing the maximum likelihood estimation (MLE) [32]. When database D consists of N samples and is expressed as $D=\left\{D_{1},D_{2},\ldots ,D_{N}\right\}$, MLE tries to find the best parameter θ by maximizing the likelihood function, l(θ|D). The log-likelihood of θ is represented as follows:

(4) $$l\left(\theta |D\right)=\log P\left(D|\theta \right)=\log\overset{N}{\underset{l=1}{\prod }}P\left(D_{l}|\theta \right)=\overset{N}{\underset{l=1}{\sum }}\log P\left(D_{l}|\theta \right)=\underset{ijk}{\sum }N_{ijk}\log\theta_{ijk}$$

where $\theta_{ijk}$ is defined as *k*th probability of a conditional probability of $P\left(X_{i}=k|\pi_{i}=j\right)$. In other words, the MLE estimate $\theta_{ijk}^{*}$ for $\theta_{ijk}$ can be calculated as follows:

(5) $$\theta_{ijk}^{*}=\frac{N_{ijk}}{N_{ij}}$$

After the model structure and the CPT of all nodes are established, the BN can be used to propagate probabilities from the root to the following other nodes under given evidence [33].

## 3. Information Value

Information value (IV) is known as a very useful concept for variable selection during the model construction in the industry. The IV helps to rank variables based on their significance for the predictive model and it can be stated as follows:

(6) $$IV=\sum^{\;}\{P\left(E|H\right)-P(E|\bar{H})\}\log\frac{P\left(E|H\right)}{P(E|\bar{H})}$$

where *H* and *E* represent the hypothesis or theory and some evidence, respectively. The negation of H is denoted by $\bar{H}$. The first term on the right, $P\left(E|H\right)-P\left(E|\bar{H}\right)$, measures the importance of deviation. The second term, $\log P\left(E|H\right)/P\left(E|\bar{H}\right)$, known as the weight of evidence (WOE), represents the deviation between distributions, which is the ratio of likelihood and is mathematically equal to the logarithm of the Bayes factor. In general, the IV values are interpreted as shown in Table 1 [34]. In this study, the hypothesis and evidence correspond to the normal condition of the system and the feature vectors that are used to diagnosis the system health, respectively.

## 4. Application: Train Door System Fault Diagnosis

### 4.1. Data Acquisition and Preprocessing

**Figure 2:**
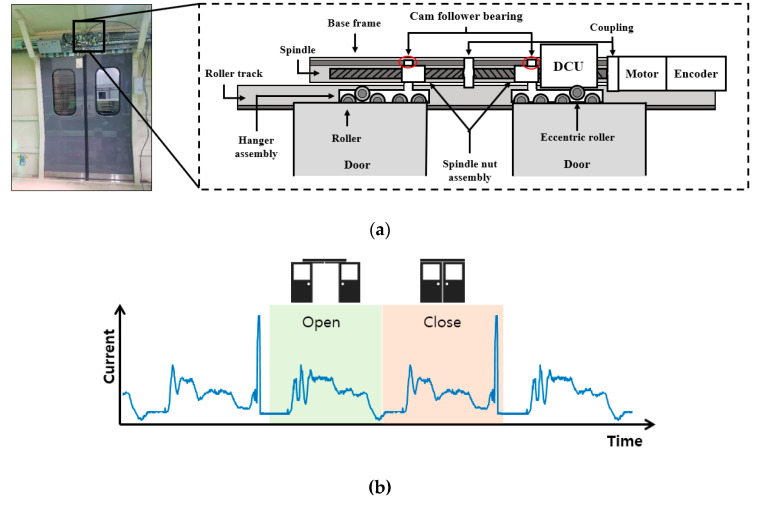

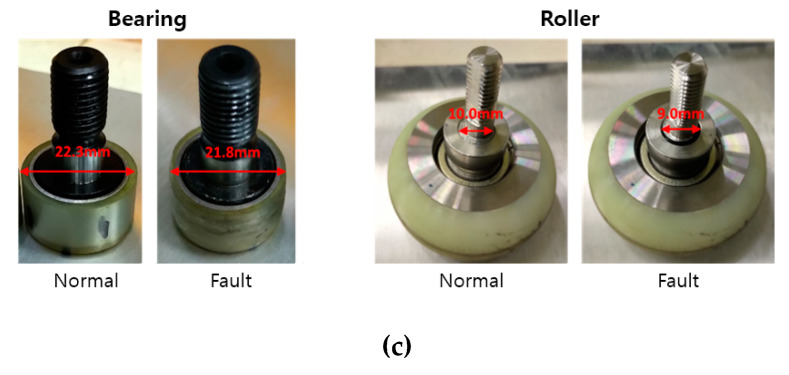
Data acquisition: (**a**) Train door system test rig; (**b**) current signal during operation; (**c**) bearing and roller specimen for test.

In this study, motor current and encoder signals acquired from the door control unit (DCU) with the sampling rates of 100 Hz and 10 Hz are utilized during the open and close operation of the train door. Figure 2a,b show the train door system test rig and the current signal obtained during the operation. In the figure, the spindle nut assembly moves along the spindle where the cam follower bearing slides within the track of the base frame is parallel to the spindle. Attached to this assembly is the hanger assembly, which hangs the door below and moves along the roller track by the rollers. Note that the eccentric roller exists inside the hanger assembly to prevent vibration during the door operation. Based on the experiences, it is known that the cam follower bearing and roller are prone to fail due to the wear. Therefore, signals are acquired for the conditions of normal and two seeded faults to the bearing and roller. The faults are shown in Figure 2c, in which the outer diameter of the bearing is reduced from 22.3 mm (normal) to 21.8 mm (fault) to induce loosening of locking, and the shaft diameter of the roller is reduced from 10.0 mm (normal) to 9.0 mm (fault) to simulate the wear between the roller and shaft. The door is operated under three different velocity conditions when it opens and closes, which are the acceleration, constant speed, and deceleration.

**Figure 3:**
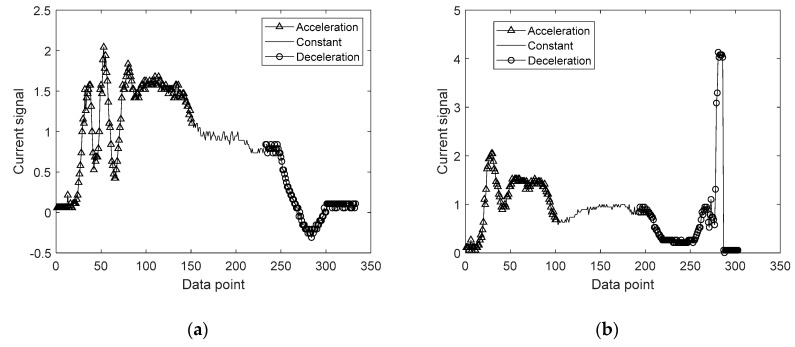
Current signal behavior: (**a**) open operation; (**b**) current signal during close operation.

The three regimes can be identified by the encoder, and the acquired signals are shown in Figure 3a,b for the open and close operation, respectively, distinguished by the symbols at each regime. For more accuracy, it is better to carry out fault diagnosis by dividing the signal into these regimes and extracting features, respectively. This is because the features can represent the condition in a certain regime more clearly, while it may not be so for the whole period. Similar attempts have been made in the literature [35,36] to cluster the data by the velocity regimes.

**Figure 4:**
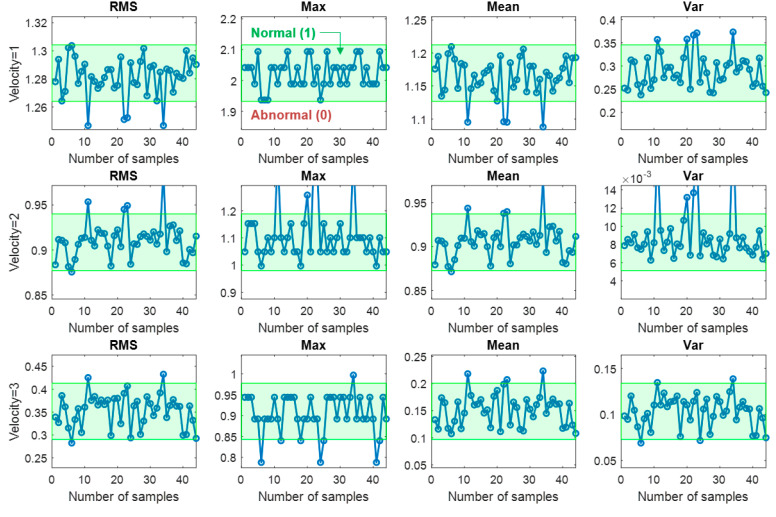
Feature transformation into the binary state during the open operation.

By considering the three regimes corresponding to different input conditions, it also makes sense to evaluate the features separately for different input conditions. Commonly used statistical features, root mean square (RMS), max, mean and variance, are extracted from each regime as illustrated in Table 2, which results in the total of 12 features. Since the BN usually deals with the discrete variables, all the extracted features are transformed into the binary states, assuming that all the features follow normal distribution, namely normal (1) and abnormal (0) where the anomaly is defined by the exceedance of 95% confidence limit. In the table, velocity regimes are labeled as follows: acceleration = 1, constant = 2, and deceleration = 3. Figure 4 illustrates the feature transformation process during the open operation. The output dataset in the database consists of six variables: one velocity state (1, 2, or 3), four feature states (1 or 0), and one door state (norm, bearing, roller). Since the number of datasets in each operation is 57, the total number of datasets for all three operating conditions becomes 171. Among them, 70% are used for the training, which is to find parameters and structure of BN, while the remaining 30% are used to test the model performance.

### 4.2. Bayesian Network Model Construction

**Figure 5:**
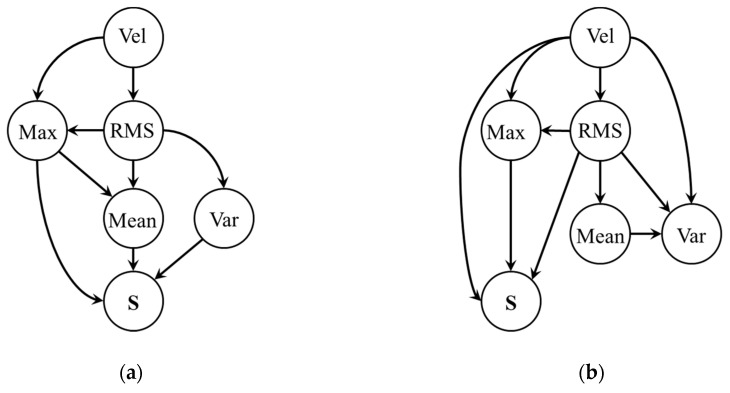
Bayesian network structure: (**a**) open operation; (**b**) close operation (Vel: velocity, Max: Maximum, Var: variance, S: door state).

As mentioned in Section 2.2, the optimum BN structure is constructed by using the K2 algorithm. The algorithm requires node ordering and the number of maximum parent orders as an important input. In this study, the velocity regimes and the door state are chosen as the root node at the top and the final node at the bottom, respectively. Node ordering is then set as: *Vel*, RMS, *max*, *mean*, *var*, *door* *state*, with the number of nodes *n* being six. The maximum number of parents *u* for a node is constrained at three to reduce complexity of the model. Using the training data, the BN structures are constructed by applying the K2 algorithm for the open and close operation as shown in Figure 5a,b respectively. As shown in the figure, different BN structures are obtained for each operation. For the open operation, door state (S) is found to have conditional dependency on the Max, Mean and Var, whereas it has the Vel, Mean and Var in the close operation. These structures represent that the door health conditions can be estimated by monitoring the condition values of these nodes. Note that the structures in Figure 5a,b are those maximizing the probability function (3). In fact, the log of the function being −512.82 at the initial structure converged to −228.5 and −255.5, respectively, at the two optimum structures. Using the constructed BN, CPTs for open and close operation are obtained next based on the MLE approach. As an illustration, CPTs of the last node, which is the door state (S), and three nodes connected with *S* are given in Table 3 and Table 4. Once the BN and CPTs are available, they can be applied to diagnose the door health condition, i.e., fault can be predicted through the belief propagation of the network. Given a velocity condition (acc’ 1, const’ 2, or dec’ 3) and corresponding state (normal 1 or abnormal 0) of each feature, the door state is predicted by the posterior probabilities for the three failure modes: normal, bearing fault, and roller fault. For example, during the close operation, when Vel, RMS, and Max are at the state 1, 0, and 0, respectively, the BN indicates that the door has the chance of roller fault with 97.78%. This can be expressed in the form of conditional probability as *P* (*S* = Roller | Vel = 1, RMS = 0, Max = 0) = 0.9778. With this information, one can estimate the health condition of the train door system. For each of the training data, the door state is predicted in this way and validated by the true state. The accuracies of the open and close operation are validated by using the training datasets and their results are 82.53 and 78.83%, respectively.

### 4.3. Fault Diagnosis Based on Information Value

**Figure 6:**
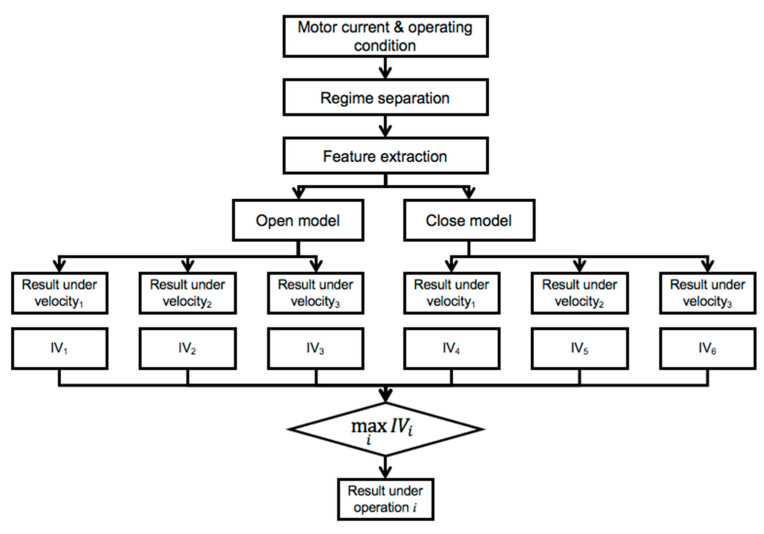
Information value-based fault diagnostics procedure

**Figure 7:**
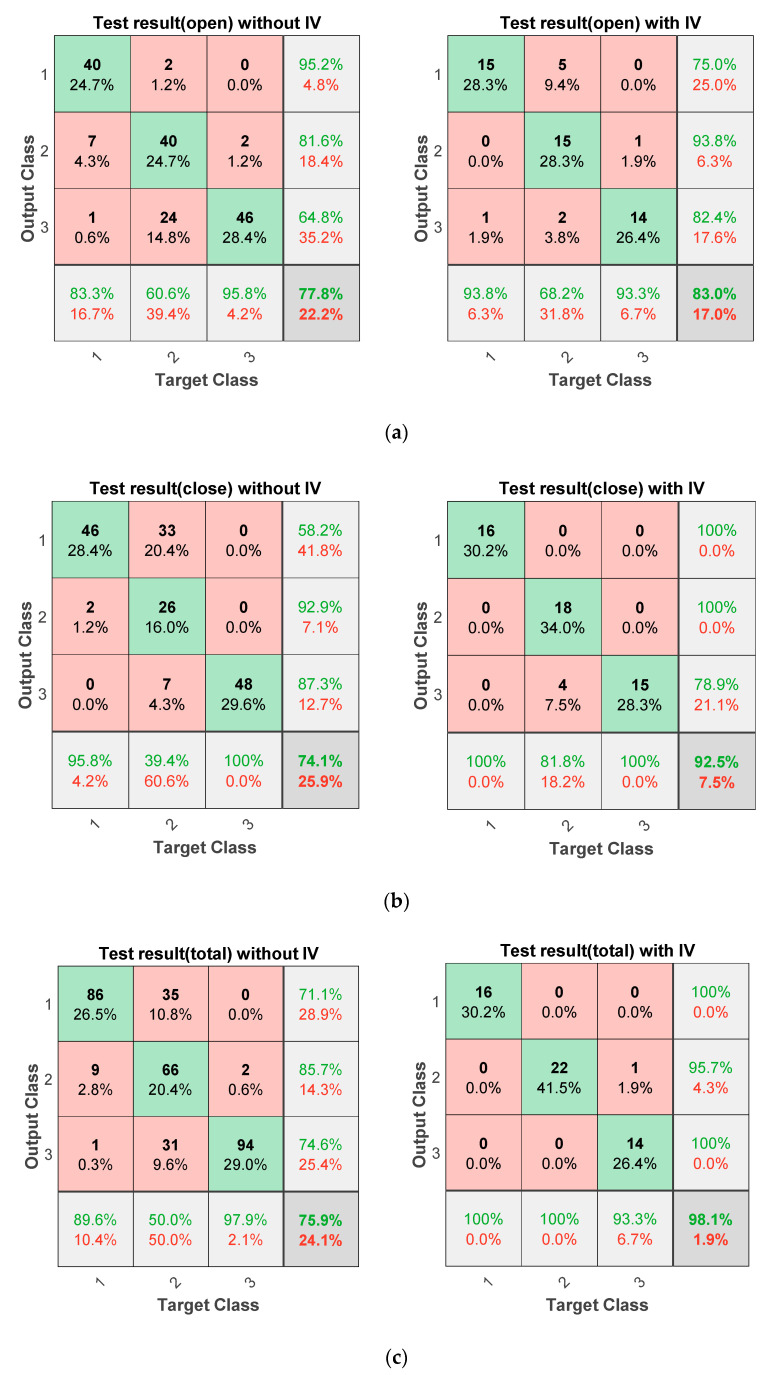
Comparison of fault diagnostic results before and after applying information value: (**a**) open operation; (**b**) close operation; (**c**) open & close operation.

As mentioned, when the system operates under different conditions and multiple diagnosis models are established for each condition, the result can be different for each operating condition. To resolve the conflicting issues in terms of diagnosis performance, one should determine which result is the most reliable. In the train door system, three different fault prediction results were obtained for three velocity conditions. As an example, Table 5 shows this problem, which diagnoses three different door conditions for an open operation. That is, the door is considered to be bearing fault at the acceleration stage (Vel = 1) and the constant speed (Vel = 2), while normal at the deceleration stage (Vel = 3). To overcome this problem, proposed information value (IV) is utilized to obtain a single door condition by following the procedure described in the Figure 6. Table 6 describes the example of IV calculation. Let *H* be the normal state, with its negation $\bar{H}$ being the other two fault states. Evidence *E* represents the variables that are directly connected with door state node. For open operation, *E* becomes Max, Mean, and Var. Based on the calculated IV in Table 6, one can recognize that the deceleration stage (Vel = 3) shows the highest IV with 0.8340, which means among three stages, the deceleration stage is the most reliable. Finally, diagnostic results from the deceleration stage are employed. The test data are used to evaluate the performance of the BN constructed by the training data, and the proposed IV-based decision-making process is applied to the BNs for open and close operations. Note that six IVs are obtained during one reciprocal operation: open and close, three for each operation. Table 7 shows the result of the IV calculation on arbitrarily chosen test data. As shown in the table, IV shows the highest value at the acceleration stage in the close operation. As mentioned, the result for the stage with the highest IV is considered to be the most reliable. Figure 7 compares the accuracy of prediction using BN with and without applying the IV by using the confusion matrix. The confusion matrix is widely used as model performance measure whose row and column represent the predicted class from a trained model and its true class. In this application, classes 1, 2 and 3 represent, respectively, normal, bearing fault, and roller fault. Their diagonal elements represent the number of records that are predicted correctly, whereas nondiagonal elements describe the number of records that are misclassified. In other words, the matrix element of *i*th row and *j*th column represent the number of samples that were classified as *i*th class, whereas their true class is *j*th class. In addition, the percentage value written below the element represents the ratio between corresponding samples and total number of samples. The percentage values colored as green and red in the last row or column represent the rate of success and fail of classification, respectively, and their summation becomes 100%. The diagonal element at the last column represents the accuracy of the model. The confusion matrix shown in the paper is constructed by using MATLAB software [7]. Note that the results without IV are those obtained for each of the three velocity stages and the highest probability is determined as a diagnostic result. Therefore, the total number at each column is three times larger than those with IV. On the other hand, the total number of test data for the results with IV reduces to one-third because only the velocity condition whose IV is the maximum among the three is used for prediction. As shown in Figure 7, after applying IV, the estimation accuracy increases during both open and close operations. In addition, test results for the case that uses the open and close operations simultaneously show the highest performance among three approaches using IV. This is because the classifier could utilize six classification results during one cycle, which means that more information can be employed to determine the door health state than other two approaches using single open or close model.

## 5. Conclusions

Fault prediction using a Bayesian network provides more information (i.e., probabilistic reasoning) for effective reasoning than a deterministic fault diagnosis algorithm. To realize effective fault diagnostics, operation conditions, such as rotating speed and loading condition, should be considered properly. For this purpose, this paper performed regime partitioning, which is widely used to deal with fault diagnosis problems under multiple operating conditions. In addition, information value was proposed to deal with the situation when multiple diagnostic results exist, which are derived from the results of each regime. Future work can be considered as two mainstreams: A continuous Bayesian network will be considered to alternate binary Bayesian networks. Even if the Bayesian network was originally developed for a binary condition, continuous versions are expected to show more accurate results. In addition, a dynamic Bayesian network will be developed to deal with real-time data.

## Figures and Tables

**Table 1:** Interpretation of information value.

Information Value (IV)	Attribute Predictiveness
Less than 0.1	Weak
0.1 to 0.3	Medium
0.3 to 0.5	Strong
>0.5	Over-predicting

**Table 2:** Feature extraction for velocity condition.

Velocity	RMS	Max	Mean	Variance
1	$RMS_{1}$	$Max_{1}$	$Mean_{1}$	$Variance_{1}$
2	$RMS_{2}$	$Max_{2}$	$Mean_{2}$	$Variance_{2}$
3	$RMS_{3}$	$Max_{3}$	$Mean_{3}$	$Variance_{3}$

**Table 3:** Conditional probability table during the open operation.

Max.	Mean	Var	Door Condition		
			Normal	Bearing Fault	Roller Fault
0	0	0	0.0333	0.2000	0.7667
0	0	1	0	0.7258	0.2742
0	1	0	0.2727	0.7273	0
0	1	1	0.1923	0.8077	0
1	0	0	0.8333	0.1667	0
1	0	1	0.2857	0.7143	0
1	1	0	0.3333	0.6667	0
1	1	1	0.9823	0.0177	0

**Table 4:** Conditional probability table during the close operation.

Vel.	RMS	Max	Door Condition		
			Normal	Bearing Fault	Roller Fault
1	0	0	0	0.0222	0.9778
1	0	1	0.1190	0.8810	0
1	1	0	1	0	0
1	1	1	1	0	0
2	0	0	0.0435	0	0.9565
2	0	1	0.3750	0.6250	0
2	1	0	0.7500	0.2500	0
2	1	1	0.5294	0.4706	0
3	0	0	0	0.1579	0.8421
3	0	1	0.0625	0.1875	0.7500
3	1	0	0.2857	0.7143	0
3	1	1	0.6308	0.3692	0

**Table 5:** Estimation result during one open operation.

Vel.	Max	Mean	Var	Door Condition		
				Normal	Bearing Fault	Roller Fault
1	1	0	1	0.2857	0.7143	0
2	0	1	0	0.2727	0.7273	0
3	1	0	0	0.8333	0.1667	0

**Table 6:** Information value calculation during one open operation.

Vel	Max	Mean	Var	P(E|H)	$P(E|\bar{H})$	WOE	IV
1	1	0	1	0.2000	0.3571	−0.5798	0.0911
2	0	1	0	0.3000	0.5714	−0.6444	0.1749
3	1	0	0	0.5000	0.0714	1.9459	0.8340

**Table 7:** Information value during open and close.

Operation	Vel	IV	Door Condition		
			Normal	Bearing Fault	Roller Fault
Open	1	0.4987	0.2727	0.7273	0
Open	2	0.0240	0.0333	0.2000	0.7667
Open	3	0.0240	0.0333	0.2000	0.7667
Close	1	0.6318	0.1190	0.8810	0
Close	2	0.0231	0.5297	0.4706	0
Close	3	0.1600	0.6308	0.3692	0

## References

[1] Lee J., Wu F., Zhao W., Ghaffari M., Liao L., Siegel D. (2014). Prognostics and health management design for rotary machinery systems—Reviews, methodology and applications. Mech. Syst. Signal Process..

[2] Pecht M., Jaai R. (2010). A prognostics and health management roadmap for information and electronics-rich systems. Microelectron Reliab..

[3] Xia T., Dong Y., Xiao L., Du S., Pan E., Xi L. (2018). Recent advances in prognostics and health management for advanced manufacturing paradigms. Reliab. Eng. Syst. Saf..

[4] Zerhouni N., Atamuradov V., Medjaher K., Dersin P., Lamoureux B. (2017). Prognostics and Health Management for Maintenance Practitioners-Review, Implementation and Tools Evaluation. Artic. Int. J. Progn. Heal. Manag..

[5] Isermann R. (2005). Model-based fault-detection and diagnosis - Status and applications. Annu. Rev. Control..

[6] Introduction to Data-Driven Methodologies for Prognostics and Health Management. https://link.springer.com/chapter/10.1007/978-3-319-55852-3_2.

[7] Kim S., Choi J.H. (2018). Convolutional neural network for gear fault diagnosis based on signal segmentation approach. Struct. Health Monit..

[8] Janssens O., Slavkovikj V., Vervisch B., Stockman K., Loccufier M., Verstockt S., Van de Walle R., Van Hoecke S. (2016). Convolutional Neural Network Based Fault Detection for Rotating Machinery. J. Sound Vib..

[9] Li C., Sanchez R.V., Zurita G., Cerrada M., Cabrera D., Vásquez R.E. (2016). Gearbox fault diagnosis based on deep random forest fusion of acoustic and vibratory signals. Mech. Syst. Signal. Process..

[10] Long J., Zhang S., Li C. (2020). Evolving Deep Echo State Networks for Intelligent Fault Diagnosis. IEEE Trans. Ind. Inf..

[11] Sobie C., Freitas C., Nicolai M. (2018). Simulation-driven machine learning: Bearing fault classification. Mech. Syst. Signal Process..

[12] Kim S., Kim N.H., Choi J.-H. (2020). Prediction of remaining useful life by data augmentation technique based on dynamic time warping. Mech. Syst. Signal Process..

[13] Turgis F., Copin R., Loslever P., Cauffriez L., Caouder N. Design of a testing bench for simulating tightened-up operating conditions of train’s passenger access. In Proceeding of the European Safety and Reliability Conference (ESREL).

[14] Cauffriez L., Grondel S., Loslever P., Aubrun C. (2016). Bond Graph modeling for fault detection and isolation of a train door mechatronic system. Control Eng. Pr..

[15] Lin S., Jia L., Qin Y., Yu B., Wang Y. (2014). Research on Urban Rail Train Passenger Door System Fault Diagnosis Using PCA and Rough Set. Open Mech. Eng. J..

[16] Dassanayake H., Roberts C., Goodman C.J., Tobias A.M. (2009). Use of parameter estimation for the detection and diagnosis of faults on electric train door systems. Proc. Inst. Mech. Eng. Part O J. Risk Reliab..

[17] Boussif A., Ghazel M. (2018). Model-Based Monitoring of a Train Passenger Access System. IEEE Access..

[18] Yan J., Lee J. (2005). Degradation Assessment and Fault Modes Classification Using Logistic Regression. J. Manuf. Sci. Eng..

[19] Cai B., Huang L., Xie M. (2017). Bayesian Networks in Fault Diagnosis. IEEE Trans. Ind Inf..

[20] Yang L., Lee J. (2012). Bayesian Belief Network-based approach for diagnostics and prognostics of semiconductor manufacturing systems. Robot. Comput. Integr. Manuf..

[21] Xu B.G. (2012). Intelligent fault inference for rotating flexible rotors using Bayesian belief network. Expert Syst. Appl..

[22] Cai B., Zhao Y., Liu H., Xie M. (2017). A data-driven fault diagnosis methodology in three-phase inverters for PMSM drive systems. IEEE Trans. Power Electron..

[23] Zheng Y., Zhao F., Wang Z. (2019). Fault diagnosis system of bridge crane equipment based on fault tree and Bayesian network. Int. J. Adv. Manuf. Technol..

[24] Zhang J. (2006). Improved on-line process fault diagnosis through information fusion in multiple neural networks. Comput. Chem. Eng..

[25] Niu G., Han T., Yang B.S., Tan A.C.C. (2007). Multi-agent decision fusion for motor fault diagnosis. Mech. Syst. Signal Process..

[26] Yan W., Xue F. Jet engine gas path fault diagnosis using dynamic fusion of multiple classifiers. Proceedings of the International Joint Conference on Neural Networks.

[27] Korb K.B., Nicholson A.E. (2010). Bayesian Artificial Intelligence.

[28] Learning Bayesian Network Model Structure from Data. https://apps.dtic.mil/sti/pdfs/ADA461103.pdf.

[29] Cooper G.F., Herskovits E. (1992). A Bayesian Method for the Induction of Probabilistic Networks from Data. Mach. Learn..

[30] Tabar V.R. A Simple Node Ordering Method for the K2 Algorithm based on the Factor Analysis. Proceedings of the 6th International Conference on Pattern Recognition Applications and Methods (ICPRAM 2017).

[31] Larranaga P. (1996). Structure learning of bayesian networks by genetic algorithms: A performance analysis of control parameters. IEEE Trans. Pattern Anal. Mach. Intell..

[32] Zhou Y., Fenton N., Neil M. (2014). Bayesian network approach to multinomial parameter learning using data and expert judgments. Int. J. Approx Reason..

[33] Huang Y., Wang Y., Zhang R. (2014). Fault troubleshooting using bayesian network and multicriteria decision analysis. Adv. Mech. Eng..

[34] Yap B.W., Ong S.H., Husain N.H.M. (2011). Using data mining to improve assessment of credit worthiness via credit scoring models. Expert Syst. Appl..

[35] Lapira E., Brisset D., Ardakani H.D., Siegel D., Lee J. (2012). Wind turbine performance assessment using multi-regime modeling approach. Renew Energy..

[36] Baraldi P., Maio F.D., Rigamonti M., Zio E., Seraoui R. (2015). Clustering for unsupervised fault diagnosis in nuclear turbine shut-down transients. Mech. Syst. Signal Process..

